# Sixteen years of eHealth experiences in Iran: a qualitative content analysis of national policies

**DOI:** 10.1186/s12961-021-00795-x

**Published:** 2021-12-11

**Authors:** Seyyed Meysam Mousavi, Amirhossein Takian, Mahmood Tara

**Affiliations:** 1 Management and Leadership in Medical Education Research Center, Kerman University of Medical Sciences, Kerman, Iran; 2 Health Foresight and Innovation Research Center, Health Services Management Research Center, Institute for Futures Studies in Health, Kerman University of Medical Sciences, Kerman, Iran; 3 Department of Health Management, Policy and Economics, School of Public Health, Tehran University of Medical Sciences (TUMS), Tehran, Iran; 4 Department of Global Health and Public Policy, School of Public Health, Tehran University of Medical Sciences (TUMS), Tehran, Iran; 5 Health Equity Research Centre (HERC), Tehran University of Medical Sciences (TUMS), Tehran, Iran; 6 Department of Medical Informatics, Faculty of Medicine, Mashhad University of Medical Sciences, Mashhad, Iran

**Keywords:** eHealth, Policy analysis, National policies, Iran

## Abstract

**Introduction:**

As a building block of all health systems and a multi-sectoral domain, eHealth has a critical role to accelerate the achievement of sustainable development goals (SDGs), particularly universal health coverage (UHC). Our objective was to provide a better understanding of the recent experiences on eHealth policy, particularly in framing process of the policies and strategies, in an attempt to develop evidence-based recommendations that can inform future eHealth policy formulation, implementation, and development in Iran.

**Methods:**

We undertook an exploratory, descriptive, comparative, retrospective and longitudinal analysis of eHealth policies by using content analysis of upstream and other key national policy documents, guided by criteria for reporting qualitative research (COREQ). A systematic and purposive search was conducted to identify publicly-accessible documents related to eHealth policies in Iran, followed by in-depth, semi-structured, open-ended interviews with purposefully identified national key informants in the field of eHealth. MAXQDA^®^ 12 was used to assist with qualitative data analysis.

**Findings:**

We retrieved and included 13 national policy documents demonstrating 16 years experiences of recorded eHealth policy in Iran, from 2004–2020. Our analysis revealed seven main categories as challenges of eHealth policies in Iran: (1) lack of comprehensive and big picture of all eHealth components; (2) lack of long-term and strategic plans on eHealth; (3) poor consistency among national policy documents; (4) unrealistic and non-operational timing of policy documents; (5) inappropriate identification and lack of involvement of key actors in development and implementation of eHealth policies; (6) low priority of eHealth in the national health system, and (7) unconventional focus and attention to Electronic Health Record (EHR).

**Conclusion:**

The findings reveal almost two decades history of eHealth initiatives at the national and upstream policy level in Iran, with noticeable gaps between desired policies and achieved expectations. The inclusion of eHealth solutions in the policy documents has been controversial and challenging. eHealth seems to have not been meaningfully established in the minds and views of policy makers and senior manager, which might have led to the development of incomplete and contradictory policies. The health system in Iran needs, we advocate, the design of an evidence-informed eHealth roadmap, as well as continuous, systematic, and reasonably time-bounded strategic plans to establish eHealth as the building block of health system along the pathway towards sustainable health development.

**Supplementary Information:**

The online version contains supplementary material available at 10.1186/s12961-021-00795-x.

## Introduction

The term electronic health (eHealth) came into use in around 2000 [1], and is defined as “use of information and communication technologies (ICT) for health [2, 3], or “an emerging field in the intersection of medical informatics, public health and business, referring to health services and information delivered or enhanced through the Internet and related technologies” [4], which is regarded as a modern driver of universal health coverage (UHC) and quality healthcare delivery [5]. The implementation and adoption of eHealth solutions, e.g., telehealth, mobile health (mHealth), telemedicine, electronic health records (EHR), electronic prescription (ePrescription) [6, 7], is increasingly being introduced to improve and strengthen the quality of care, medical error, patient safety, while also reducing healthcare expenditure [8–13].

There is an increasing interest to use eHealth solutions to help professionals improve their relationship with patients as well as better and faster patients’ access to healthcare services and strengthen the health systems [7, 14, 15]. In addition, through improving the allocation of resources, planning, cost-efficiency, better provision of healthcare services, timely monitoring and evaluation, preventive measures, and personalized services, eHealth can facilitate policy-making processes [16, 17]. eHealth not only can play a key role in structural reforms, which are necessary for the sustainability of health systems, but also has an important effect on reliable and safe access to healthcare services [18–20]. It is worth noting that large-scale and national-wide initiatives are implementing in various countries to coordinate eHealth policies and this trend is expected to continue [21]. Let alone COVID-19 pandemic will enhance the extra complexity that health systems are facing, due to the interconnected and multidimensional characteristics of healthcare services [22].

In Iran, the utilization of eHealth solutions has become more popular among national health policymakers during the past two decades. Mainly as a result of improved access to basic information and technological advances, different eHealth programs have been designed and implemented across the country. Clear understanding of how eHealth policies were formed and evolved in Iran can inform legislative and policymakers at various levels for consistent policy making, while it also can guide financial investment towards advancing various types of eHealth solutions throughout the country.

At the international level, several organizations periodically published their reports in eHealth including but not limited to World Health Organization (WHO), International Telecommunication Union (ITU), and International Organization for Standardization (ISO) [2, 3, 23–26]. In these reports, in addition to providing some successful experiences or assessing the current situation of countries in eHealth policies and implementation, they also provide practical and policy recommendations and suggestions, which could be potentially useful for national policy makers.

This article, as a part of a broader, multi-phase, and mixed-methods national study, aims to explore the 16 past years of eHealth experiences in Iran. It offers a broader historical overview of relevant policies, critically discusses the approaches taken, and highlights the strengths as well as possible areas for improvement. Our findings will contribute, we envisage, to a better understanding of the recent experiences on eHealth policy and will provide evidence-based insights into the framing of the policies and strategies in the field. As a robust eHealth infrastructure can establish the backbone of sustainable health development, our analysis can assist developing more effective recommendations to inform future eHealth policy formulation, implementation, evaluation and development in Iran, and perhaps similar settings.

## Methods

### Design

We undertook an exploratory, descriptive comparative, retrospective and longitudinal analysis of eHealth policies by using qualitative content analysis of key national policy documents.

### Data collection

Policy documents are defined as written documents that contain strategies, priorities, and proposed measures, along with defining objectives and goals, drafted by the legislative/governmental/consultative/executive organizations. In order to identify all national policy documents related to eHealth, we comprehensively searched databases of related organizations including the Ministry of Health and Medical Education (MoHME), Ministry of Information and Communication Technology (MICT), Ministry of Cooperatives, Labour, and Social Welfare (MCLSW), and Islamic Parliament Research Center in Iran. Further, to enhance comprehensiveness of the search and by using purposive sampling technique, we sought some key informants’ opinions in identifying national documents. Policy documents were eligible for inclusion if they were related to eHealth policies/programs/projects/implementations, focused on national level, and considered eHealth in their content. The inclusion and exclusion criteria were defined through a participatory approach with consultation of selected national key informants. A pre-defined form was used to extract legal/policy issues related to eHealth. A member of the research team (SMM) was responsible for an in-depth review of all policy documents.

To better analyze and interpret the content of policy documents, the research team conducted in-depth, face-to-face, semi-structured, and open-ended interviews with 13 national key informants in the field of eHealth to supplement with policy documents analysis (Table 1). We adhered to the consolidated criteria for reporting qualitative research (COREQ) guidelines [27]. The COREQ checklist can be found in Additional file 1. Our research team developed a pre-pilot interview guide, which was used to conduct the interviews. It should be noted that some minor revisions and changes were made in the interview guide following the initial interviews. The key informants were identified using a combination of purposive and snowball sampling techniques [28] to include eHealth senior experts who were well-familiar with the process of formulating policy, and had considerable experience in designing and approving the related policy documents. The recruited participants had excellence working experiences as national managers, policy advisors, university professors, private sector advisors, CEO of private and knowledge-based companies. All interviews were conducted by first author (SMM). Interviewees did not have any personal relationship with the study team. The study objectives and reasons for doing the research were provided and reiterated to the interviewees prior to each interview. Interviews lasted 55 min on average and were conducted in a time and location convenient for the key informants. The recruiting process was continued when saturation was achieved, i.e. when no relevant new data raised [24, 29]. All interviews were digitally recorded and transcribed verbatim. If necessary, the interviewer took notes.

**Table 1:** Characteristics of the national key informants

Title	Education	Gender	Experiences and involvement in eHealth policies
P1	PhD in Health Information Management	Male	Academic and project coordinator
P2	MD	Male	National manager
P3	MD & PhD in Health Informatics	Male	National manager and policy advisor
P4	MD	Male	CEO of private company
P5	MD & MPH	Male	National manager and policy advisor
P6	PhD in Community Medicine	Male	Policy advisor
P7	PhD in Medical Informatics	Male	Academic and policy advisor
P8	PhD in Health Information Management	Male	Academic and policy advisor
P9	PhD in Healthcare Services Management	Male	National manager
P10	MSc in Management of Information Technology	Female	National manager and project coordinator
P11	PhD in Future Study	Male	CEO of a knowledge-based company and policy advisor
P12	PhD in Health Policy	Male	Academic and policy advisor
P13	MD and PhD in Healthcare Services Management	Male	Academic, policy advisor and private sector advisor

Data obtained from interviews and policy documents were considered as valid sources with equal value. While policy documents were used as a basis for identifying arguments of the analysis, interview data were used to exemplify, illustrate, or juxtapose the findings obtained from policy analysis of documents [30].

### Data analysis

We used the content analysis approach for qualitative data analysis. Initially, all documents were carefully read and reviewed to achieve immersion. The characteristics of the related policy texts were identified through inference based on both manifest (explicit) and latent meaning (implicit). For each document, phrases, sentences, and paragraphs related to the subject were extracted and categorized. Notes and data tables were used to compare documents. Short summaries of the findings were extracted from the data tables and the comparisons were combined. Besides, aspects of the related data were read, extracted and noted.

To analyze the interviews, one researcher (SMM) listened to the audio files and read and reviewed the transcripts several times to achieve immersion, until the semantic units were identified. In the next step, the semantic units were summarized to become more abstract and coded. Phrased expressed by interviewees were used to select the codes. The codes were compared in terms of similarities and differences, and were categorized into some subcategories and categories, which were then discussed and finalized by the entire research team. SMM extracted and analyzed the data independently, under the supervision of AT. We tried to resolve any discrepancies in data analysis through internal discussion, with arbitration by the third member (MT) if we could not reach an agreement. A qualitative content analysis adopted from Elo and Kyngäs [27], which facilitates the analysis of large volumes of textual data and different textual sources was used in the analysis. Three main phases of analysis included: preparing, organizing and reporting [27]. We used MAXQDA^®^ 12 (VERBI software GmbH, Germany) to assist with qualitative data analysis. Finally, the research team merged the key findings and concepts to establish interpretations and provide a final report.

### Ethical approval

This study received ethical approval from the Ethics Committee for Research at the Tehran University of Medical Sciences (IR.TUMS.SPH.REC.1396.3998).

## Results

After a thorough review of national policy documents, 13 documents were found as eligible for inclusion. The list of documents as well as their details are provided in Table 2.

**Table 2:** The list of included national policy documents

Document	Approved by	Approved/published year
Constitution of the Islamic Republic of Iran	The assembly of experts for the constitution and approved by referendum	December 1979
The 4th Five-Year National Development Plan Act	The Parliament	September 2004
Enactments of the 6th Meeting of the Supreme Council for Health and Food Security	Supreme Council for Health and Food Security	January 2009
Enactments of the Cabinet	The Cabinet of government	May 2010
Comprehensive scientific map of Iran	Supreme Council of the Cultural Revolution	January 2011
The 5th Five-Year National Development Plan Act	The Parliament	January 2011
Comprehensive Scientific Map of the Health Sector	Ministry of Health and Medical Education	October 2012
Health System Transformation Map	Ministry of Health and Medical Education	2011/October 2012
Mega Policies on Health	Developed by Expediency Discernment Council and decreed by the supreme leader	April 2014
Instructions, responsibilities, and authorities of the Electronic Health Services Working Group	The Supreme Council of Information Technology	January 2015
Enactments of the Supreme Council of Cyberspace	Supreme Council of Cyberspace	April 2016
The 6th Five-Year National Development Plan Act	The Parliament	March 2017
Enactments of the 15th Meeting of the Supreme Council for Health and Food Security	Supreme Council for Health and Food Security	December 2017

We extracted seven main categories from our qualitative content analysis of policy documents. In this section, we do not separately mention and describe each of the categories, rather we tried to present the results in an integrated way. To begin with, we describe the details of eHealth related policy documents.

### Constitution of the Islamic Republic of Iran

The constitution was approved as a result of a referendum in 1979, which was amended during another referendum in 1989. Article 2 of the Constitution has paid special attention to the advancement of sciences, techniques, and experiences. Information and communication technology (ICT) is considered a key strategy to facilitate the achievement of human goals and welfare. eHealth is one of the key applications of ICT in the current world, whose essential role in improving population health, enhancing quality of healthcare services, reducing medical errors, and improving the efficiency and effectiveness of healthcare interventions is well-proved.

Article 29 of the Iranian Constitution states:

“It is a universal right to benefit from social security in respect of retirement, unemployment, old age, disability, being stranded, absence of a guardian, accidents, and from health and medical services and care provided through insurance or other means”.

Therefore, providing healthcare services is a universal right, to which the government is responsible and accountable. Currently, the use of electronic solutions for health insurance, mHealth, electronic health records (EHRs), etc., is fundamental to fulfil Article 29, meaning the use of advanced technologies to improve community health, as emphasized by the Constitution.

### The fourth five-year national development plan act

The Act was passed on September 2, 2004, to be executed during the period 2005–2009. The parliament decided to extend it for another year until 2010. This Act was one of the first upstream policy documents that emphasized eHealth explicitly. According to Article 88, the MoHME was obliged to “design and establish a comprehensive health information system for all Iranian citizens”. This policy is intended to continuously improve the quality and excellence of health services, increasing productivity, and optimal use of health facilities in the country.

One interviewee, while mentioning this Act, noted that this plan mentioned one of the components of eHealth for the first time:

“I'm going to mention an issue that is of crucial importance for the eHealth in Iran. I mean it has directed this field. I think for the first time the fourth national development plan mentioned eHealth. … citizens’ health should be electronically.”

 (P1)

In contrast, another interviewee criticized the Act:

“One of the turning points was the Article 88 of the Fourth Plan Act, which mentioned EHR or eHealth as a form of smart card … That view was wrong, which means you can't lunch EHR only by using smart card.”

 (P2)

### Enactments of the sixth meeting of the supreme council for health and food security (SCHFS)

The SCHFS is led by the president and its secretariat is coordinated within the MoHME. It is Iran’s governance mechanism to foster health in all policies and materialize effective multisectoral collaboration for health [31]. On January 19, 2009, the SCHFS, in line with Article 4 of its enactment, urged the MoHME, in cooperation with the Ministry of Cooperatives, Labour, and Social Welfare (MCLSW); the Ministry of Information and Communications Technology (MICT); the Supreme Council of Information Technology (SCIT); the Supreme Council of Informatics; and the Forensic Medicine Organization; to develop the operational plan and executive regulations for the creation and development of EHR (comprehensive health information system of citizens) within one year. The aim was to create appropriate information platform for providing novel services to citizens within a 10-year horizon. Let alone, according to Article 88 of the 4th National Development Plan Act, a comprehensive health information system (HIS) for the entire populations should have been designed and established by the end of this program. Nevertheless, this Article (Article 4) replaced the previous one and defined a new 10 year horizon for eHealth in Iran.

In 2008, the Cabinet approved a bill, which in line with Article 4 of the SCHFS Act, mandated the MoHME to develop an operational plan and executive regulations for the creation and development of EHR (comprehensive health information system of citizens) in cooperation with relevant ministries and organizations. While Article 88 of the 4th National Development Plan Act asked for a comprehensive system of citizens' health information to be established by the end of 2009, the SCHFS’ new bill urged the MoHME to formulate the operational plan and executive regulations of this system only, which made it impossible to implement and adapt a comprehensive EHR by the end of 2009.

### Enactments of the cabinet

On May 22, 2010, prior to the approval and implementation of the 5th National Development Plan Act, the Cabinet introduced the development of eHealth and e-welfare as one of the country's main priorities in the field of ICT. In addition, the MoHME was tasked to develop universal EHR system for the whole population within the next five years, which contradicted the previous Cabinet enactment that set a 10 year horizon for this target. Therefore, as most interviewees highlighted, the MoHME failed to reach the goal mainly due to insufficient basic infrastructure and inadequate legal support for EHR.

“How many laws do we have in the field of eHealth? All the related infrastructures such as law of privacy, law of data exchange system, law of data and EHR availability and access, law of electronic signature of documents, law of electronic financial documents, none of them have started…”.

 (P3)

“The Takfab plan… immediately targeted the EHR. Targeting is great. The MOHME want to launch the EHR in the country… .. It has a base pyramid, you build a pyramid from scratch or you have to build it from the base? Any document you are reading/reviewing in eHealth, this pyramid sets a rule, infrastructure + standards. [Foreign consultant came to Iran] We said, sir, the ministry wants to do this, he said you can build the pyramid from the top, but everywhere in the world when you build a pyramid from the top, it becomes unstable, he said your project fails”.

 (P4)

### Comprehensive scientific map of Iran

The Supreme Council of the Cultural Revolution (the body appointed by the Supreme Leader to make and oversee upstream educational and scientific decisions) approved the comprehensive scientific map of Iran on January 4, 2011. The map categorizes the country’s science and technology priorities into three categories of A, B, and C, based on their importance (A the highest). Category A emphasizes ICT and other priorities related to the health sector, although eHealth is not mentioned. In category B, only “health information and knowledge management” is highlighted among priorities related to the health sector, which can be considered as an example of eHealth.

In the section on national strategies and measures for the development of science and technology, clause 11 of macro strategy “directing the cycle of science and technology and innovation to play a more effective role in the field of medical sciences and health” is related to the health sector.

In this macro strategy, the national strategy of “development and localization of health sciences and technologies in the country” has been introduced. The national measures related to this macro strategy are “development of ICT in the field of health in order to establish an eHealth system in accordance with the Islamic ethics, social security, and privacy”. It is worth noting that this is the first upstream policy document that has explicitly mentioned the eHealth system.

One key informant mentioned that this document is not systematically linked to other policies:

“In fact, in my view, there is no comprehensive and integrated plan with systemic links with other programs”.

 (P5)

### The 5th five-year national development plan act

The Act (2011–15) was passed on January 15, 2011. Similar to the 4th plan, the Parliament also extended its execution for another year, which lasted until 2016. As policy makers’ insight about ICT was gradually enhancing over time, this Act consists of more legal items about HIT, i.e., Article 35 with direct and Articles 46, 48, 54, and 211 with indirect link to eHealth.

Article 35 mandated the MoHME to establish the Iranian EHR system and information systems for health centers in line with the National Database of the Statistical Center and the National Organization for Civil Registration (NOCR) of Iran, aiming to maintain integrity in the knowledge management and information in the health sector. This Article has also emphasized on the privacy and confidentiality of information as well as prioritizing the family physician and referral system program. Moreover, all health centers, both governmental and non-governmental, have been obliged to collaborate with the MoHME in this regard. Article 35 also obliged the MCLSW to organize health insurance services in an integrated and IT-based manner through the implementation of the Iranian EHR system, in collaboration with health insurance organizations and healthcare providers, within the first 2 years of the act (i.e. by mid-2013). All relevant organizations and institutions, both governmental and non-governmental were mandated to collaborate.

Despite the 4th National Development Plan, which emphasized designing and establishment of a comprehensive and universal HIS for the entire Iranian citizens, the 5th National Development Plan was retrograding and mandated the MoHME to establish the Iranian EHR system and the information systems for health centers. Our interviews with key informants revealed the reason behind this was that the 5th National Development plan endorsed the EHR system called SEPAS (the national hospital-based EHR), which was developed by the MCSIT as an middleware with the main goal of connecting hospitals and medical centers as well as aggregation of medical information at the MoHME level. Such application was far below the eHealth application highlighted by the 5th development plan, meaning development of a comprehensive EHR.

Furthermore, some interviewees pointed to the conflict of interest among some high-level officials in the enactment of this Act:

“They were all looking for SEPAS. The same thing that they had designed as an electronic health record system, I remember that in the drafts, even in parentheses, they wrote the SEPAS; Now they have to say why they wanted to do such a thing.”

 (P6)

“The technique layer has a role in preparing it, the fifth and sixth plan Act. Who do you think writes this in their draft? you know what I'm saying.”

 (P7)

While other interviewees highlighted insufficient infrastructure as a reason:

“One of the main reasons for the failure in achieving some goals of the fifth development plan is the lack of sufficient financial resources. I mean we developed a plan, but we did not receive any money, while both MoHME and Planning and Budget Organization were involved. We underestimated the required budget and they did not allocate this insufficient budget.”

 (P2)

The MCSIT, both in the 5th and 6th national development plans, developed the instructions on EHR, which were not finalized in the 5th plan for the following reasons:

“We sent the developed instructions, based on the fifth national development plan, to the Planning and Budget Organization. They reviewed the instruction several times in the Social Commission. However, conflicts were raised with health insurance organizations.”

 (P2)

Although the 6th National Development Plan put this item on the agenda, it is still under review by the Law Office of the MCSIT.

### Comprehensive scientific map of the health sector

The MoHME has developed and published the comprehensive scientific map of the health sector in October 2012. Based on the country’s comprehensive scientific map. Indeed, the MoHME is among the first ministries that developed such scientific map, which should be revised every three years. Nevertheless, no revision has happened so far.

Concerning the “desirable status of indicators”, we can only refer to a “10% reduction in health care costs using the modern technologies and sciences, which mainly intends to reduce inpatient costs”. This indicates the extent to which technology might affect cost reductions. Ten percent is a symbolic number and is used to increase the understandability of this concept.

Regarding the priorities of health sciences and technologies, there is only one item that is related to eHealth, under the banner of “health information and knowledge management”. In the section on policies, “increasing the capacity of knowledge production”, “disseminating and sharing the produced knowledge”, and “facilitating and establishing communications” are emphasized as two related strategies.

Concerning the development of human resources, we can mention “strengthen the association between … health sciences and ICT”, which resembles attention to education and inclusion of ICTs into the curriculum of health sciences disciplines.

In the section on “actions**”** and subsection of development of policymaking, management, and laws, “defining an organizational structure for health information technology centers in all organizations” has been mentioned. In the section on increasing the knowledge production capacity, “designing a comprehensive system of diseases and risk factors using information technology (by emphasizing the use of geographical information system (GIS), remote control, and long-term prediction of health consequences of climate changes)” and “creating information banks in high priority areas (with the ability to share information)” have been mentioned. In the section on the development of human resources, only “creating a comprehensive network of electronic education and health decision-making for public utilization and service provider groups” have been mentioned.

In the section on requirements, issues such as “revising the law on intellectual property and copyright (the requirement to comply with its various treaties)” have been emphasized, which require drafting and approval of laws in accordance with the conditions of the society and the increasing importance of ICTs. We could not find any item on confidentiality, security, safety, and privacy. Also, the law on the necessity of formulating priorities and annual programs of science, technology, and innovation in MoHME and the requirement for its public release” has been noted, which concerning the eHealth field, such issue is rare. In addition, the Law on the “provision of statistics, information, and performance of governmental institutions on an annual series” has been also proposed as a requirement, which has been rarely occurred in the field of health, similar to the previous issue. As a result, periodic assessments are either not implemented or their information are not publicly available. Another requirement is related to the “law on sharing information of governmental organizations with public and private research institutes”.

Concerning the “monitoring and evaluation indicators”, the issues related to the eHealth field include the following indicators: “approval and application of laws related to intellectual property rights that are implementing”, “the number of online distance medical education courses and modular curriculums”, “the percentage of ICT-related resources to all financial resources of the health system”, “number and percentage of hospitals with electronic medical records (separated by outpatient and inpatient services)”, “internet penetration rate in various regions of the country”, and “public education about the importance and solutions of science development, technology, and innovation”.

An interviewee, who participated in the drafting sessions of this map, mentioned:

“I was in a meeting. The ministry had learned to gather expert opinions through brainstorming. The concepts weren't defined, without clarifying the concepts for all members, everyone was commenting (some were complicated, and some were weak). There were significant differences, I don't know how they are going to pool the opinions to develop a scientific plan for the MoHME. I was insisting that before brainstorming, first, you have to define the basic concepts. You should say, Mr. X, the definition of EHR is this, as there are various definitions. Therefore, the output of the brainstorming was not homogeneous. And I think the final plan cannot be considered as a scientific plan, but it was introduced as a scientific development program.”

 (P8)

One of the national managers said that the document was not comprehensive enough:

“The scientific map of the health sector was presented ... they were boasting that we have developed the scientific map of the health sector. But it is not comprehensive enough at all. A part of this plan is on IT ... But it is different from what it has to be, although long-term plans don't go deep in details.”

 (P9)

### Health system transformation map

This document was developed in 2011 and published in October 2012. It is stated that this roadmap is in line with the Islamic-Iranian progress model as well as the comprehensive scientific map of the country. The document is introduced as a remedy that if implemented properly, it can bring valuable progress in the field of health, as endorsed in the national vision statement of 2025. In the section on “macro goals”, there is no item on eHealth. The section on “policies” contains 15 items, whose last one is on eHealth: “developing IT for the promotion of health programs”. In section of “National Transformation Programs”, there are national plans for each of the 15 policies, and all national programs contain focal points.

The 15th policy item contains three plans on “development of basic electronic services in the health system”, “equal access to resources and health services using the ICT”, and “quick, inexpensive, sustainable, and secure access to health records of all people in the community using ICT”. In addition to the 15th policy, which is directly related to eHealth, there are also national programs related to eHealth in other policies. In the sixth policy (“comprehensive and integrated health care with a focus on equity and emphasis on accountability, transparent information, effectiveness, efficiency, and productivity in health networks in accordance with the referral system and the family physician program in both urban and rural areas”), one of the national transformation plans is “development of Telehealth care services”.

The 12th policy (“implementation of a health observatory system, strengthening the supervision and reporting system, monitoring and evaluation of both inter-and-intra sectoral programs of the health sector”) has also emphasized “designing a comprehensive system on diseases and risk factors using ICT.

### Mega policies on health

Decreed by the supreme leader on April 7, 2014, this upstream policy document contains 14 main policies, is in line with the constitution (paragraph one of Article 110). The initial draft of these policies was mentioned in the health system transformation map that was developed by the Expediency Discernment Council. The initial draft contained a policy on “developing ICT for the promotion of health programs”, which was removed in the final policy document, as one interviewee wonders about its reason:

“Initially, there was a policy on eHealth, but, unfortunately, it was removed in the process of formulating and approving Mega Policies on Health.”

 (P6)

### Instructions, responsibilities, and authorities of the electronic health services working group

In its eighth session on January 12, 2015, the SCIT approved the instruction on formation, responsibilities, and authorities of the Electronic Health Services Working Group. This instruction is intended to enhance coordination, synergy, and unity among responsible organizations through integrating information and knowledge into the development of eHealth plans, based on an agreement among the MICT, the MoHME, and the MCLSW. The main objectives are to increase control over the process of expanding infrastructures and determining and explaining the role of key stakeholders in order to strengthen the leadership in line with upstream policies of information technology in the country.

The eight main strategies are as follows: (1) improving information integrity; (2) comprehensive access to stakeholders; (3) creating intelligent health information management tools; 4) developing security and confidentiality of information at all levels and value chains; (5) designing health information architecture; (6) managing and sharing health information; (7) standardization of electronic health services and solutions, and (8) continuation of electronic health services. The main purpose of formulating these strategies was to design and develop all activities and programs that are designed, approved, and implemented in the country, by taking into account the mentioned strategies.

### Enactments of the supreme council of cyberspace

On April 3, 2016, the Supreme Council of Cyberspace (SCC) approved that the Supreme Council of Informatics and Council on Security of Information Exchange to be dissolved and all of their strategic, policymaking, supervision, and coordination duties to be transferred to the SCC at the national level. Moreover, the Supreme Council of Information Technology was renamed as the “Executive Council of Information Technology” and all its strategic tasks in the fields of setting policies for monitoring and coordination were transferred to the SCC. This council operates within the framework of the general policies of the country and enactments of the SCC.

In addition, in line with Article 2, it was emphasized that the responsibilities of the Executive Council of Information Technology should be transferred to the SCC. However, the eHealth working group was not transferred to other councils and has still remained as a part of the Executive Council of Information Technology. It is worth noting that the last meeting of this council was held on 28 October 2015, which was before the dissolution of the Supreme Council of Information Technology.

### The 6th five-years national development plan act

The Act was approved by the Parliament on March 4, 2017 (2017–21). Although there were many similarities with the 5th plan, the 6th plan put more emphasis on eHealth. In Paragraph A of Article 74, the MoHME is obliged to establish the Iranian EHR system and information systems for health centers within the first two years of this Act (i.e. by the mid of 2019) in coordination with the National Database of the SCI and the NOCR, while paying special attention to the privacy of information, the family physician program and referral system. Moreover, within 6 months after the full establishment of the above system, in collaboration with health insurance organizations and healthcare centers, the MoHME was mandated to organize health insurance services based on ICT and in meaningful interaction with the “Iranian EHR” system. This Article requested other eHealth applications to be in harmony with the MoHME. In other words, similar to the 4th national development plan, the MoHME was recognized as the custodian of eHealth. Paragraph E of this Article emphasized that implementation of the comprehensive and universal health services system is a multi-dimensional task that comprises of multiple applications, including the Iranian EHR. In Paragraph C of this article, health insurance organizations and funds were obliged to move towards strategic purchasing of health services only through the Iranian EHR System.

According to Paragraph H of Article 68, the MICT should establish eHealth applications that cover all beneficiaries until the end of the sixth plan. One interviewee was of the view that the legislator tried to implicate its political will in this Article:

“If you look at the sixth plan, MICT is obliged to support two areas, especially concerning eHealth, taxation, and so on. So all the attention in the political sector is going this way.”

 (P3)

Looking at Paragraphs A and E of Article 74 reveals an ambiguity whether the MoHME is the sole steward of the “Iranian EHR System” and “information Systems of health centers”. However, Article 68 of this Act introduced the MICT as the steward of eHealth, as an umbrella for EHR!

In addition, Article 74 obliged the MoHME to establish the “Iranian EHR system” and information systems of health centers. This issue is not sufficient according to one national manager who thought that the legislator should have specified the executor of this Article, or at least the MoHME, as the steward of health sector, should have the authority to determine the executor:

“When according to the law an issue should be implemented, the executor should also be determined by the law. For example, it should be clear that which organization should implement the EHR system? According to the law, the MoHME is in charge of this issue. The MoHME should select the executor. Who is the executor? A few months ago, we gathered here for developing the executive regulations and we said, Sir, please specify the executor. After several meetings, they are going to select the executor still”.

 (P3)

It took 1 year and a half until the MoHME selected an individual, not an organization, to conduct the implementation of the plan:

“For example, concerning the EHR, a month ago, Mr. X, who was the deputy director of the Information Technology Organization, was transferred here. Now, he is the deputy of our center, Mr. Y's successor. The minister selected him as the executor of the EHR”.

 (P10)

In other words:

“For Article 74 of the Act, minister has selected Mr. X”.

 (P2)

This selection was heavily criticized as this person had little (if no) experience in the field of eHealth. In other words, the process of selecting the executor was extremely contested, while established organizations were overlooked and an individualistic approach which was not in line with professionalism was taken:

“In my opinion, an institution or organization should be in charge of this responsibility (e.g. MCSIT). Or at least a secretariat, namely EHR secretariat, should have been established.”

 (P10)

Another national manager described the appropriate process of implementing these legal issues:

“This executer should develop the instructions. Then, it should be confirmed by the Cabinet. Then, a line should be embedded in the annual budget of the next year. Afterward, the budget should be confirmed by Parliament. Then, the budget should be allocated. After that, the supreme audit court will monitor how the budget is spent. When none of these will be implemented, so what are we looking for?!”

 (P3)

On the other hand, Paragraph A of Article 74 asked actors (i.e. SCI and NOCR) to collaborate with the MoHME for implementing the Iranian EHR system. This issue can be due to the legislator's serious weakness in recognizing the key and influential actors in the field of eHealth. Not only the legislator/policymaker's had poor understanding about EHR and its applicability to improve quality of care, but important stakeholders including the MICT, the ICT guild organization of Iran, and National Standard Organization (NSO) were not engaged enough in the process. eHealth capacity therefore became as an instrument to produce statistical reports only, for instance through the use of EHR. As a result, various national development plans endorsed these articles repeatedly, without a meaningful implementation of eHealth happening in reality:

“This project has not been productive. They cannot be able to start another stage, while the previous stage is not over yet. It is expected that this process will be continue again.

 (P8)

Nevertheless, some interviewees rejecting the repetition:

“No, this is not repeated. If you look at it again, you can see some changes. The issues that have caused this law not to be implemented have been amended. For example, previously Article 35 contained Paragraphs A and B, but currently, these two are merged into Paragraph A. In other words, previously there were Paragraphs for creating insurance systems and EHR systems. These are somehow necessary, if the insurance system is not formed, you cannot easily run the electronic records. What did the legislator do? Integrated two paragraphs in a particular Article! Currently, the MoHME is introduced as the steward of the system, as previously no one was in charge of this system.”

 (P2)

Another national manager mentioned that senior managers like to use “ex post facto” to implement the plans. In other words, their tendency is towards abiding the law:

“The health sector is wide-ranging. For example, when we talk about the infrastructures of eHealth programs, it contains several issues e.g., according to the fourth and fifth plans, we connected all public hospitals using a web-service. But after a while, someone will say, it only contains financial data, and there is no clinical data. We have to collect clinical data. But it’s a large-scale project with several processes. First, the necessary information should be collected, then it should be designed and piloted. Afterward, we can use it national-wide. When you are now this middleware is ready, but the amount of stored data is growing, which means its coverage is expanding in different areas. Concerning its repetition in the law, I think we're going to use the executive coercion of the law. We want to inform our healthcare providers that this is the law and must be observed. But if we refer to the fifth plan, they would say it's over. Then, it should be emphasized by the six (National Development) plan, and establishing EHR is continuous”.

 (P10)

Many interviewees mentioned that EHR is a process, not a project, for which an implementation timeframe might be inappropriate. Rather, awareness and commitment in this regard are fundamental missing characteristics in this process:

“We didn't play a role in this process at all. I mean we didn't propose this law, even, its draft.”

 (P3)

Some interviewees reminded in order to build a robust eHealth, the big picture must be painted initially, followed by embedding its components within the law, the status that has not occurred so far:

“Stewards such as the minister should emphasize this issue when the law is developing. They should invite various actors and ask them to draw the big picture. For example, this part should be implemented in the fourth plan, this part for the fifth, and this for the sixth plan. As such a big picture is not developed and because these issues require a deep understanding. They only discuss macro issues and are interested to generally mention, but if they leave it details, they cannot put anything else on it according to their priority.

 (P10)

### The 15th meeting of the SCHFS

The SCHFS meeting in December 2017 in the presence of the President approved the establishment of a technical committee within the Secretariat of the SCHFS to review the trends as well as opportunities and threats of using digital technology in the health sector. The MoHME became responsible to set up the committee in coordination with the Vice President for Science and Technology and the MICT. The Committee was mandated to provide a report in the next meeting:

“When we talk about a pathology document, it should contain recommendations. Or, for example, a time frame would be set to achieve the goals.”

 (P6)

One interviewee pointed to a close collaboration between Vice President Office for Science and Technology and the MICT in drafting the Paragraph:

“To somehow, we have developed this Paragraph, how? We (Deputy for Science) performed a review about digital health advancement in the world, which attracted Vice President for Science and Technology, and he provided the report to the President on same day. The president said that if I wanted to do this, this should be presented to the SCHFS. Then, the Vice President presented this issue to the council in 30 minutes. The council asked us to evaluate the opportunities and threats of digital health, so we began the project”.

 (P11)

Another interviewee portraited the process of selecting digital health as follows:

“According to the National Foresight Program that is confirmed by the Cabinet in March 2015, the deputy for scientific affairs is in charge of this issue. It has a policy-making deputy, which has the main responsibility. They established a committee and decided to only focus on three programs (i.e. health, transportation, and energy). Then, they said the health sector is so wide, you should specify specific themes. We formed a team of few experts and decided to focus on the digital health field.”

 (P11)

Currently, more than 3 years has passed since the adoption of this enactment, but not only the annual meeting of the SCHFS has not been held, but also no specific intervention has been implemented concerning this legislation:

“I don't know what the Secretariat of the Council is doing. They sent a letter to the Vice President for Science and technology after months of delay. The MICT, MoHME, and IT department were asked to introduce a representative to form a committee to investigate this issue. When we initiated this project, I had a letter that introduced me as the representative to the Secretariat of the SCHFS. … They thanked me and said, you are the first institution that introduced a representative, while the MCSIT of the MoHME did not introduce its representative”.

 (P11)

Reviewing the above policy documents revealed the weakness of planning and lack of a big picture of all eHealth solutions to move forward:

“It has been treated like an add-on, an uninvited child ..., which means we have considered eHealth with various understandings; about infrastructures, so it's not satisfying at all.”

 (P12)

All documents that we reviewed, we only came across the macro issues, perhaps because:

“The more general the mentioned issue, the higher would be our flexibility to categorize the previously conducted programs as a part of the policy, which means receiving more budget. But, if you make them more specific, for example, ePrescription. I cannot develop an electronic system for laboratories. Also, I cannot receive budget for this item. The same would be true for human resources. Hence, the more general the policy, the more flexibility we will have. Generally speaking, it is true for all policies. If they are going to mention details, they will add some clauses.”

 (P10)

One major problem of upstream policies as excessive focus on EHRs (as just one of eHealth's examples):

“Many of our upstream policies did not mention eHealth, rather they only focused on EHR.”

 (P12)

“We do not have a general law for eHealth. I didn't hear. These issues are mentioned in the national development plan, which is temporal. The 6th plan mentioned developing a system, named SEPAS, if they are going to continue its development. They have to make it a law.”

 (P6)

One interviewee pointed out to the reasons for this issue:

“The technique layer also has its supporters. You think who has developed the fifth and sixth plans. You know what I mean. Initially, they developed a draft in the ministry. Then, they send the document to the integration commission for further discussion. They add something. As I said we have EHR and the Iranian electronic health records system. These systems are mentioned in the economic, political, and social development plans of the country. If you can implement them perfectly, you can be happy.”

(P7)

Another interviewee confirmed this issue and noted that:

“The EHR is a minor component; it's more about policy than technical issues. While it has to define the policy or set the targets that the government should expand electronic services to about 80 percent of urban and 50 percent of out-of-town activities in health sector.”

 (P13)

However, one of the interviewees defended the inclusion of instructions related to EHR in the law and emphasized that:

“In that development plan, their focus is on EHR. For example, it also contains other issues such as mobile phones or things like that. That's normal, because EHR is the foundation of all electronic services. I mean, if you don't have EHR, you cannot focus on other services. I mean you cannot focus highly. When you have EHR, you can develop other options, the same as other countries. EHR is the foundation.”

 (P1)

Another interviewee also pointed to another point about embedding some legal issues:

“So who made this? Look, there are many things that I cannot mention. However, I know the answer. Anyway, I was a member of the team that made these decisions. The decisions are based on some efforts performed by a group or institution. We know the process of developing the law. It's not as you think, at all.”

 (P7)

One MCSIT manager confirmed this issue and noted that:

“As you know, Lobbyists have an important influence over drafting a law. Everyone is seeking his/her interests. Lobbying is common in many countries and is not solely related to Iran. For example, some use lobbying to cancel an enactment that is developed by a particular group. Or initially, you support a program, then you tell your friends to cancel the program”.

 (P10)

Therefore:

“For eHealth as an overall concept, I've never seen any policy... About the general concept of eHealth, in fact, we do not have any regulation, instruction, or rule … Nevertheless, it's emphasized by the policy paragraphs (i.e. the fourth development plan, the fifth, and the sixth plan).”

 (P13)

## Discussion

To the best of our knowledge, this is the first study that has used the content analysis approach for analyzing national eHealth policies in Iran. Our findings revealed that due to the expansion of ICT, the Iranian policymakers and legislators have paid special attention to eHealth during the last two decades. However, some fundamental barriers hindered the effective and meaningful progress in the implementation and adoption of eHealth products in the Iranian health system.

Most importantly, there has been no reliable big picture for required eHealth components and their implementation priority in the form of a national policy in Iran. In the era that all countries with advance use of eHealth paid a particular attention to robust strategic plans for HIS [5, 10, 13, 32], no national strategic plan has been developed in this field, which led to a series of challenges and obstacles for eHealth policies and programs in the country.

Our findings also identified the lack of consistency among some national upstream policies in the subject of eHealth. For example, the fourth National Development Plan Act emphasized on the establishment of the “comprehensive health information system for all citizens” until 2010. By enacting this law, the SCHFS attempted to change the legislation by emphasizing on the development of EHR until 2018. Besides, it has considered EHR as a comprehensive health information system. Whereas, in another inconsistent decision, the Cabinet emphasized developing EHR for all citizens until 2015. On the other hand, according to this enactment as well as the enactment of the SCHFS, while comprehensive health information system for all citizens and EHR are two completely different terms, they were considered identically equivalent. This inconsistency might indicate two fundamental issue. First, if the legislator has mentioned a program in the fourth NDPA and further decided to change it, it should have been confirmed by the Parliament. It is worth noting that the enactments of the SCHFS and the Cabinet cannot fill this legal gap. Second, it is not clear as why EHRs and comprehensive health information system for all citizens were not synergistically developed, while policymakers diverted their attention towards developing EHR.

As another example, we can mention the comprehensive scientific map of the country and the scientific map of the health sector. While the former is the first national policy that mentioned eHealth as a necessity, the latter did not mention this key issue at all. Unrealistic and non-operational timing of proposed policy documents is another important problem. For example, several national policies set different schedules for the implementation and establishment of EHR. Both the 5th and 6th NDPA emphasized on implementation of this program in two years (2013 in the fifth NDPA and 2017 in the sixth NDPA). Another important issue is the schedule set by the Cabinet, which emphasized that a universal EHR for the entire population should be a quantitative target for the MoHME within the next 5 years (2015). It is worth noting that national implementation of the EHR is a complex and time-consuming process [33]. In Singapore, a pioneer country in implementing eHealth programs, since 2004 public hospitals are sharing patients' data electronically. Meanwhile, their policy-makers expect full implementation will take another 5–10 years [34]. In contrast, while patients' data, as one of the most basic measures in the field of EHR, are not shared electronically, the legislator expects to achieve the universal implementation of comprehensive eHealth within two years.

Our analysis also revealed inappropriate identification and lack of meaningful engagement with key stakeholders in definition and implementation of eHealth policies. For example, the 5th and 6th NDPAs mentioned the SCI and NOCR as the key identified stakeholders of EHR, who were obliged to cooperate with the MoHME, both of whom were meaningfully involved in the process of policymaking for eHealth. Another example was the enactments of the Cabinet and SCHFS, which referred to the Forensic Medicine Organization as a key actor. This can be due to the legislator/policymaker’s incomplete understanding of two key issues: (1) the concept of eHealth; and (2) determining key actors involved in the process.

Another crucial problem was policy-makers’ inadequate insight and knowledge about eHealth and its priority to improve healthcare quality and safety. As an example, “Mega policies for health” did not mention eHealth at all. WHO considers information systems as one of the six building blocks of any health system. Let alone, according to the WHO recommendations, the implementation and adoption of eHealth can accelerate achieving UHC, reducing healthcare expenditure, improving quality, and enabling equitable access to healthcare services [30]. The WHO also acknowledges that countries seeking UHC, should invest in and support eHealth [31]. Over the past years, Iran has designed and implemented a series of reforms such as Health Transformation Plan (HTP) to reduce OOPs and improve the quality and access to healthcare services with the aim of achieving UHC [35]. The legislator/policymaker's understanding of eHealth was mainly related to EHR, which has led to ignoring other important eHealth solutions, such as telemedicine, mobile mHealth, ePrescriptions, and so on. In addition, the implementation of EHR requires several infostructures, i.e. data interchange interoperability and accessibility; privacy, security, and safety regimes; consent, access control and workflows; clinical terminologies and classifications; identification registries and directories; census information, population information, and data warehouse; and standards, all of which must be considered by the policymaker.

Although EHR is considered as one of the components of infostructure, this does not indicate its dominance over other programs. Let alone many policy makers in Iran consider EHR mostly as patient administration system (PAS), a very basic component of comprehensive EHR. The challenge of distributing medicines, which is currently a national priority, can be used as an example here. ePrecription is one eHealth component that can help policymakers/managers regulate and implement appropriate distribution policies for medicines. While Iran is suffering from massive unilateral and unfair sanctions by the United States, which has been exacerbated by the COVID-19 pandemic [36], shortage of medicines is a serious problem. Nevertheless, some experts think that the problem is not the shortage of medicines, rather it is related to the distribution, which can easily be addressed by eHealth solutions like ePrescription. Many countries have enacted laws and implemented policies to promote the widespread use of ePrescription [37]. It is well-recognized that ePrescription has a great potential and can play a key role to track and manage the distribution and allocation of drugs, prevent and control unnecessary demand, appropriate supply of services and care, improve the quality of care, prevent unnecessary costs due to readmission of patients, and prevention of non-urgent visits.

## Conclusions

eHealth has a 16-years history in national policy documents in Iran. This study provided some fresh insights into the status of recent experiences on eHealth policy in Iran. We found that the inclusion of eHealth solutions in the policy documents has been controversial and challenging, e.g.: (1) lack of comprehensive and big picture of all eHealth components; (2) lack of long-term and strategic plans on eHealth; (3) poor consistency among national policy documents; (4) unrealistic and non-operational timing of policy documents; (5) inappropriate identification and lack of involvement of key actors in development and implementation of eHealth policies; (6) low priority of eHealth in the national health system, and (7) unconventional focus and attention to EHR. eHealth seems to have not been meaningfully established in the minds and views of policy makers and senior manager, which might have led to the development of incomplete and contradictory policies at the national level. In the aftermath of COVID-19 pandemic and along the pathway to rebuild its health system to reach sustainable health development, Iran needs to redesign the roadmap for eHealth mega picture, in which main real actors are involved and contextual issues have been carefully considered. Moreover, eHealth should be re-considered as a building block of the health system and importance of this issue should be reflected in upstream national policy documents. In addition, we require a robust and concrete continuous educational system with focus on senior managers and policy makers, so to make them acknowledge the fact that the EHR is just one example of eHealth solutions, not the whole. We also advocate pragmatic and grassroot solutions to minimize “conflict of interests” when relevant authorities and senior managers make unreasonable decisions in preparing and formulating further eHealth policies.

Our analysis can assist national policymakers to inform future eHealth policy formulation, implementation, evaluation and development in Iran, and perhaps similar settings, especially other LMICs. eHealth solutions are at the heart of efforts to reach sustainable health developments anywhere, i.e. to reach UHC and enhance quality of care. Hence, design, implementation and adoption of effective reforms that boost the availability, utilization, and adoption of meaningful eHealth solutions is the key towards sustainable healthcare systems of future. As different countries are running various national eHealth policies within their own settings, where contextual characteristics play a fundamental role in the direction of such interventions, evidence-based case studies like our research can pave the way to learn from comparable settings to boost the required political commitment and effective policies to foster eHealth policies for public good.

## Supplementary Information

**Additional file 1.** The consolidated criteria for reporting qualitative research (COREQ) checklist.

## Data Availability

Not applicable.

## Abbreviations

SDGs: Sustainable Development Goals
UHC: Universal Health Coverage
EHR: Electornic Health Record
eHealth: Electronic Health
ICT: Information and Communication Technology
NOCR: National Organization for Civil Registration
mHealth: Mobile Health
MoHME: Ministry of Health and Medical Education
MICT: Ministry of Information and Communication Technology
MCLSW: Ministry of Cooperatives, Labour, and Social Welfare
SCHFS: Supreme Council for Health and Food Security
SCIT: Supreme Council of Information Technology
HIS: Health Information System
SCC: Supreme Council of Cyberspace
NSO: National Standard Organization
NDPA: National Development Plan Act
PAS: Patient Administration System
ePrecription: Electronic Prescription
MCSIT: Management Center of Statistics and Information Technology
